# Developing Action Plans in Youth Photovoice to Address Community-Level HIV Risk in Rural Malawi

**DOI:** 10.1177/1609406920920139

**Published:** 2020-04-23

**Authors:** Saria Lofton, Kathleen F. Norr, Diana Jere, Crystal Patil, Chimwemwe Banda

**Affiliations:** 1 College of Nursing, Health Systems Science, University of Illinois at Chicago, IL, USA; 2 College of Nursing, Department of Women, Children and Family Health Science, University of Illinois at Chicago, IL, USA; 3 Kamuzu College of Nursing, University of Malawi, Blantyre, Malawi

**Keywords:** photovoice, community-based research, action research, social justice, arts-based methods, PAR—participatory action research

## Abstract

Youth-driven approaches to HIV prevention can engage youth and improve health outcomes. Photovoice has been used to engage youth in identifying the assets and challenges in their communities. In sub-Saharan Africa, youth remain vulnerable to HIV infection. This article describes a photovoice project, named Youth Photovoice, conducted in rural Malawi, which focused on community places and situations relating to risky sexual behaviors that place youth at risk of HIV infection. Twenty-four youth, ages 13–17 (12 males and 12 females), participated in Youth Photovoice. During the photovoice process, youth identified five community situations and places that put them at risk of unsafe sex and thus HIV infection: initiation ceremonies, isolated places, community celebrations, local businesses such as bars and rest houses, and church-sponsored activities. Youth used a systematic action planning process to develop action plans. They presented their action plans to local leaders and parents. Parents and leaders responded positively and agreed to help the youth carry out their plans. If their plans to address community situations that put them at risk of unsafe sex succeed, there will be a direct impact on reducing the risk of HIV infection among youth. Youth Photovoice provided the opportunity for youth to obtain new skills, build new partnerships, and present their ideas to community leaders. Integrating this action planning process into photovoice helped to guide the youth toward actualizing their HIV prevention plans in their community. This process can increase the effectiveness of photovoice initiatives to address other community issues in a wide variety of settings.

Effective HIV prevention for youth is an urgent global health issue, and reducing risky sexual behaviors is one approach to addressing HIV infection rates. Youth remain highly vulnerable to HIV infection, especially in sub-Saharan Africa where 66% of all new infections occur (Avert, 2018a). In this article, risky sexual behavior is defined as unprotected sex (without a condom), sex with multiple sex partners, or sex in a high-risk context such as when alcohol is involved. Although Malawi remains one of the 10 countries with the most severe HIV epidemic globally, significant progress has been made. Malawi is on course to achieve the global 90–90–90 goals of increased testing, treatment, and viral load suppression by 2030 (The Joint United Nations Programme on HIV/AIDS [UNAIDS], 2019). However, the UNAIDS Malawi report (2019) notes that youth are being left behind. Although only a fifth of the population is 15–24 years old, a third of all new HIV infections occur in this age-group (Avert, 2018b).

A growing body of research documents that youth-led programs, including those that target bullying, healthy eating, and HIV risk reduction, have demonstrated positive changes (Connolly et al., 2015; Frerichs et al., 2015; Maticka-Tyndale & Barnett, 2010). Particularly with HIV risk reduction activities, researchers found that youth have demonstrated positive changes in condom use, knowledge, and changing community norms (Maticka-Tyndale & Barnett, 2010). Consequently, there is a global push to involve youth in community mobilization efforts to address HIV infection rates. Even so, youth-driven approaches are infrequently used in HIV prevention programs for youth (Reed et al., 2014; UNAIDS, 2018).

One strategy to increase youth-driven approaches is photovoice. Photovoice is a visual, community-engaged, participatory action research method that allows participants to express themselves visually and articulate their community’s assets and challenges when the participants analyze the photographs they have taken (Liebenberg, 2018; C. Wang & Burris, 1997). The participants then communicate their analyses to relevant policy makers with the goal of achieving community change (C. Wang & Burris, 1997). Indeed, photovoice is recommended as a strategy to engage youth participation and to initiate community mobilization (C. C. Wang, 2006). However, engaging youth participation to initiate community mobilization can be challenging. Integrating action planning, which is a detailed plan outlining specific steps, into photovoice has the potential to enhance the photovoice process and empower community members. This article describes how youth in this project, named Youth Photovoice, utilized photovoice along with action planning to identify and develop solutions to community-level HIV risk.

## Background

Photovoice has the potential to play a key role in the development of youth-driven projects (Lofton & Bergren, 2019) by providing opportunities for youth to make valuable and meaningful contributions to the community at large. As a type of community-based participatory research, photovoice allows youth to take ownership over the research by (1) empowering youth to identify and reflect on community assets and challenges, (2) encouraging critical dialogue, and (3) creating opportunities to develop a plan to inform and reach those with power (C. C. Wang, 2006).

Although photovoice has been widely used with both adults and youth, it strongly resonates with youth because its visual approach is developmentally appropriate, and the use of technology is engaging. Photovoice has the potential to provide youth with an opportunity to use photographs to reflect on their community in an open discussion and freely articulate their beliefs. Middle adolescents have demonstrated the cognitive capacity to engage in photovoice due to the rapid growth in their prefrontal cortex (Collins & Steinberg, 2006; Sanders, 2013; Vaughn et al., 2013; Woods-Jaeger et al., 2013). In particular, middle adolescents’ increased information processing, abstract thinking, self-reflection, problem-solving, logical reasoning, and executive functioning are key social cognitive developments that serve youth participants well while engaging in the photovoice process (Collins & Steinberg, 2006).

The photovoice approach has been used with youth in several African rural and urban settings. Issues addressed included a range of health and community issues such as safety (Suffla et al., 2012), HIV and AIDS stigma (Moletsane et al., 2007), overall community assessment (Dakin et al., 2015; Mmari et al., 2014), and water and sanitation (Bisung et al., 2015). There have been three community-based projects using photovoice in Malawi. One addressed resilience related to poverty as part of a cash transfer project (Barrington et al., 2017). Another explored adolescent well-being through youth daily activities (Zietz et al., 2018). The third focused on well-being and palliative care to show the utility of photovoice in palliative care research (Bates et al., 2018).

However, a gap in photovoice research is the lack of studies that use this approach to examine community factors affecting youth sexuality. No published studies have used photovoice with youth in Africa to identify factors driving the risky sexual behaviors of youth. An extensive search found only four photovoice studies globally that focused on the sexual risk behaviors of youth (Gubrium & Torres, 2013; Markus, 2012; Vaughn et al., 2013; Woods-Jaeger et al., 2013). In North Carolina, the participants provided critical reflections on relationships between the local context and sexual health (Woods-Jaeger et al., 2013). Gubrium and Torres (2013) explored the double standards in sexual communication among Latino American youth. In Wyoming, researchers examined healthy relationships among American Indian youth residing in a rural community (Markus, 2012). In New Guinea, researchers explored factors that influenced youths’ health and their perceptions of HIV (Vaughan, 2010). Given the importance of sexuality as a major component of youth development, photovoice research that focuses on the community context of risky sexual behaviors of youth in Malawi and other high-prevalence countries can make an important contribution to public health.

Another gap in photovoice research is that there are no studies about how to implement a systematic action plan to reach those in power. Photovoice projects that aim to reach policy makers generally fail to include a systematic action planning development process. Reaching those in power is an important goal in photovoice, although it is not always achieved. A systematic action plan can facilitate reaching those in power. A few photovoice projects have identified solutions and have held policy makers accountable for their plans (Rhodes et al., 2008; Sanon et al., 2014; Teti et al., 2018). For example, in the study of Rhodes et al. (2008), the participants living with HIV challenged the public health department to engage individuals living with HIV in their programming efforts. The public health department responded by partnering with the participants to develop a speaker’s bureau and to take responsibility for restocking a portion of the department’s free condom distribution sites (Rhodes et al., 2008). However, even projects that focus on identifying solutions and working with policy makers to implement solutions have rarely described the processes used to get to action plans (Rhodes et al., 2008; Sanon et al., 2014; Teti et al., 2018). Integrating action planning with photovoice can be a key strategy for the implementation of youth-developed solutions (Miller & Vaughn, 2015).

In this report, we describe how the Youth Photovoice pilot addresses both gaps. This Youth Photovoice pilot was added to an ongoing community HIV prevention project where community members are taking over implementation of an evidence-based peer group intervention to both adults and youth in rural Malawi (Jere et al., 2018). During peer group sessions with youth, the youth mentioned many community factors that contributed to risky sexual behaviors among youth. Photovoice offers an opportunity to add youth-driven community-level solutions to the ongoing implementation project focused on individual-level HIV prevention. Therefore, the research team and community decided to collaborate to pilot this youth-driven approach.

The purpose of the Youth Photovoice pilot was for Malawian youth to (1) identify and critically examine how places and situations are either protective or facilitative of engaging in risky sex and (2) develop action plans to reduce these risks and present the plans to the local community.

## Materials and Methods

### Site and Sample

Malawi has a population of 17 million, 62% of whom live on less than US$1.25 per day (World Bank Group, Malawi, 2018), and is divided into three regions—North, Central, and South. This study took place in the Phalombe district in the southern region of Malawi. In the southern region, HIV prevalence is over 15%, substantially greater than the national prevalence of 10% (Malawi Population-Based HIV Impact Assessment Project, 2016). Phalombe is a rural agricultural district with a population of 429,450. The study site is located in a community with a population of 110,623.

The facilitators for the Youth Photovoice pilot consisted of a team of U.S. and Malawian coinvestigators and community adult volunteers. This team combined prior experience with the photovoice approach and deep familiarity with the cultural, social, linguistic, and economic context and the process of conducting community-based research in the Phalombe district, Malawi. The three volunteers were all members of a community-based organization (CBO) that supports local activities for youth. The CBO had collaborated with the implementation project and was eager to support Youth Photovoice. The three volunteers had substantial experience leading youth activities in the community. Two of the U.S. investigators (S. L. and C.P.) had prior experience facilitating photovoice with youth in the United States, while the other coinvestigators had many years of experience conducting community-based research with both youth and adults in Malawi. The coinvestigators with photovoice experience provided training about photovoice, and the team met before and after each session to debrief and discuss strategies to increase youth engagement and to help youth express their ideas.

To recruit participants for Youth Photovoice, the CBO volunteers made announcements in surrounding communities to inform youth about the project and invite them to a meeting. The coinvestigators met with the 24 youth who came to the meeting. The facilitators explained the study and its eligibility criteria (between the ages of 13 and 17 years and residing locally in the community). All 24 youth were eligible and wanted to participate. Literacy was not an eligibility requirement, but all those recruited to the study were literate. Today, over 97% of Malawian youth aged 15–19 years have attended school (National Statistical Office, Malawi, 2016). The team described the project, confirmed eligibility, and answered any questions. The youth then read the consent out loud verbatim, assented to participation, and took a consent form home to their parents. Each returned the signed parental consent form the following day. The average age of the youth was 14.8 years, and half of them were male and half female. All were currently in school. Twenty-three youth attended public schools and one attended private school. Additional characteristics, such as socioeconomic status, were not collected. One youth did not return after the first day. The remaining 23 youth attended every session.

### Procedures

The youth participated in a total of eight photovoice sessions that followed the photovoice process for youth as described by C. C. Wang (2006). Youth were divided into four same-gender groups of six, and each group worked with two adult facilitators. We used same-gender groups because the research team’s previous work in Malawi found that young women are more hesitant to speak in a mixed-gender group (Kaponda et al., 2007). Sessions were held at either the CBO’s community center or a nearby public school. The eight sessions followed the photovoice procedures described by C. C. Wang (2006). The youth then developed themes using pile sorts and began to move through the action plan development process. The sessions, action plans, and presentation of the plans and photographs to local leaders and parents are discussed in detail in Results section.

In the introduction to photovoice, the facilitators discussed ethical considerations with the youth. The facilitators emphasized not taking photographs that show identifiable persons and obtaining permission for photographs of someone’s property. If any discussion or incident suggested that a youth was in a potentially abusive situation, we planned that one of our team members who is a psychiatric nurse (D. J.) would provide counseling and any needed follow-up. However, no such incident occurred. The group then discussed safety, emphasizing the importance of avoiding situations that could threaten their safety and taking pictures in pairs.

### Data Collection

The primary data presented here are the photographs the youth selected, the co-created analysis of those photographs, and the detailed action plans that the youth presented. All photographs taken by the youth and photographs of the action plans were logged, and the action plans were translated into English. Additional data were collected by research team members who each attended all sessions for one of the four groups and by data clerks who worked on the implementation study. Data collectors were trained in field observations before the photovoice sessions. After each session, the coinvestigators and data collectors met to discuss the session and review the notes. Each of the group discussions was audio-recorded, transcribed, translated from Chichewa to English, and reviewed for accuracy by our Malawian bilingual team members.

### Data Analysis

The initial data production and analysis proceeded in a three-step process: selection, contextualization, and codifying of ideas (C. Wang & Burris, 1997). First, the youth *selected* the photographs that they deemed most pertinent to the discussion. Then they *contextualized* the photographs through their discussions. Also, throughout the photovoice process, youth analysis of the photographs moved from concrete and descriptive to a more in-depth analysis linking descriptions more abstractly to the community context. Finally, the youth *codified* their ideas through the pile sorting activity, which allowed them to identify themes and rank these in terms of priorities. They then used a systematic action planning process described in Results section to develop an action plan for each prioritized theme. The themes, photographs, and action plans were then exhibited and presented orally to the community. The presentation was the product of the youths’ data production and analysis process.

In addition to the co-created analysis, the coinvestigators used data from the field notes, debriefing discussions, and transcripts of each group discussion to describe the photovoice process. These data were placed in an Excel spreadsheet and reviewed by the team to identify commonalities and differences related to gender, age, and the different groups. The photographs taken by the youth and translated action plans were linked to this Excel file so they could be incorporated into the analyses. A future publication will focus on analyses of the session transcripts and field notes.

## Results

The Youth Photovoice pilot took place over eight 2-hr sessions (16 hr of group activities plus the hours each pair devoted to taking pictures). Table 1 summarizes the focus of each group session and the strategies used at each stage of analysis. Youth presented a total of 128 photographs that captured places and situations that encouraged and discouraged risky sex. They identified that ceremonial rituals, dances, and sporting events were associated with situations that encourage risky sexual behavior. They described how isolated places, rest houses, and bottle (liquor) stores were places that encourage risky sexual behavior. Youth described schools, health facilities, and local youth organizations as places that discourage risky sexual behavior. Youth noted that church and its associated activities could be associated with both the discouragement and encouragement of risky sexual behavior. Using the photographs as triggers, each session led to a deeper discussion about the meaning of the photographs. The discussion moved from description toward a more critical analysis of situations and places represented. The eight facilitated discussions, described in detail below, allowed youth to identify the issues and possible solutions; these solutions culminated in concrete action plans.

### Session 1: Introduction to Photovoice

The research team provided a detailed overview of photovoice and the specific purpose of Youth Photovoice. The facilitators reviewed how to use smartphones and gave the youth time to practice using the phones. Next, the purpose of the photovoice assignment was discussed in more detail. In order to present the photo assignment, the facilitators placed this photo assignment in the context that risky sex puts youth at risk of acquiring HIV. Then youth were asked to take photographs of places or visually “represent” situations that encourage or discourage engagement in risky sex. The facilitators then guided a discussion about ethical and safety considerations. Youth were paired and given the smartphones so that they could go out into their community and begin to document these situations or places.

### Sessions 2 and 3: Photograph Discussion

The youth came into the second session with their first round of photographs on their smartphones. Initially, the youth and facilitators reviewed the photographs to ensure that the youth understood the photo assignment. Using the SHOWeD method (C. Wang & Burris, 1997), facilitators led youth discussions about the photographs. The SHOWeD questioning technique used to discuss the photographs asks the following: “What do you See here? What is really Happening? How does this relate to Our lives? Why does this problem or strength Exist? What can we Do about it?” (C. C. Wang, 2006). The discussion was initially slow. As the facilitator asked the youth questions, the youth would go around the circle, pair by pair, answering the questions in a succinct and direct manner. This initial slow flow of discussion was expected, for only a few of the youth knew each other before this project.

For Session 3, one of the research team members printed the photographs for the discussion; however, the youth preferred using smartphones instead. The facilitator asked each pair to answer a series of questions. The youth would pass the smartphone around or hold the phone up to show which photograph they were referring to in their discussion. With growing familiarity with one another, the youth began to provide answers and engage in back and forth discussions. Facilitators paid attention during discussions to ensure that all youth were participating in the groups. Initially, there were concerns that the youngest participants would not be able to participate fully; however, they were also able to provide just as many insights into photographs that were metaphors or symbolic representations of issues around risky sexual behavior as the older youth were able to provide.

### Session 4: Theme Development

The goal of this session was theme development using the pile sort technique (Necheles et al., 2007; Yeh et al., 2014). For this meeting, the printed photographs from the previous session were brought to the discussion. Each group of six sorted only the photographs for their group. Each group of six youth was then asked to collaboratively sort the group’s photographs into a cluster of related photographs and, through consensus, assign each cluster a name. They then wrote each cluster’s name on a large sheet of paper and glued associated photographs to the paper. They wrote details about how and why the place or situation was related to risky sexual behavior, allowing each cluster to become a theme.

**Figure 1.**
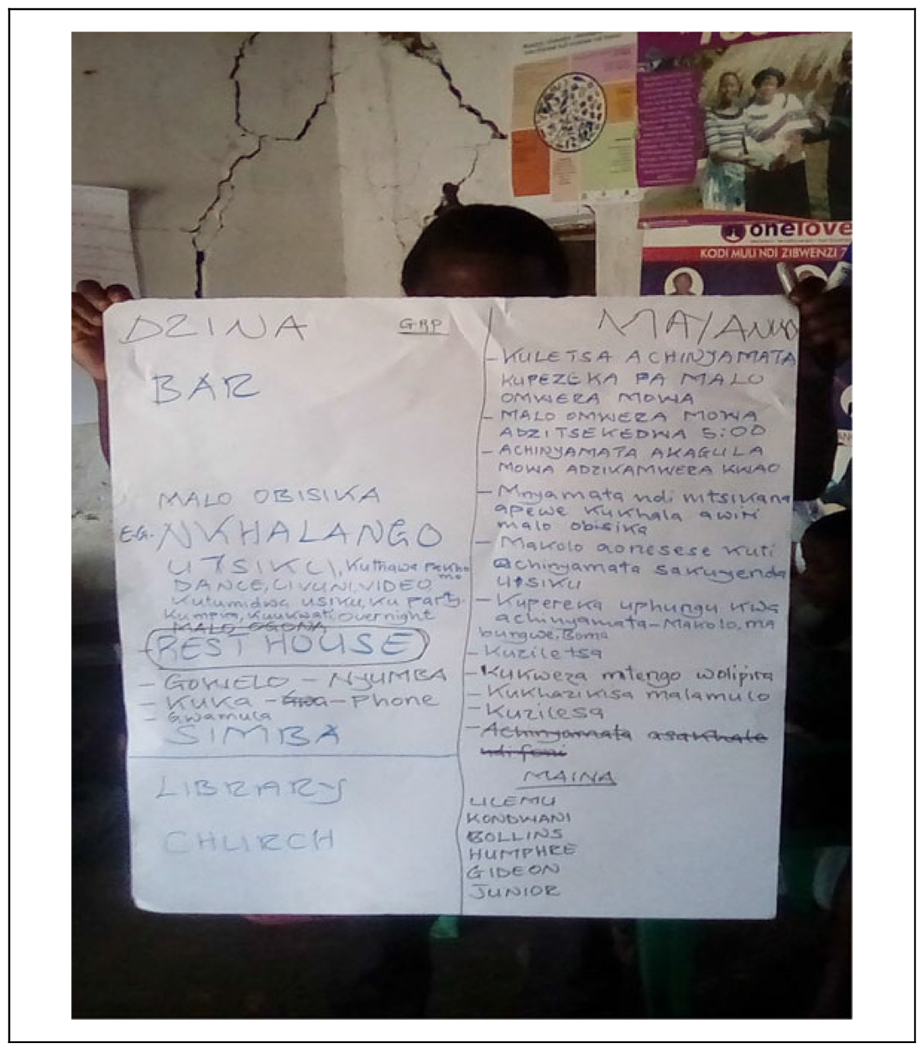
Potential problems on the left and potential solutions on the right.

The youth then worked on problem-solving and tried to identify one solution to reduce risk related to each cluster (Figure 1). The youth ranked their clusters and identified the most pressing issue and related solution(s). Guidance from the facilitators ensured that each subgroup picked a different theme. However, because one subgroup could not prioritize just one theme, a total of five major themes and solutions were identified. The youth then discussed how they would implement the solution, laying the foundations for the development of an action plan.

### Sessions 5 and 6: Action Plan Development

The facilitators had intended to introduce action planning to the youth that could be implemented in the community after the presentation (Table 2). However, they realized that the original approach was inadequate because it did not provide direction or a framework for the process. The lead investigator, who was familiar with action planning in community development, realized that the University of Kansas Community Toolbox, which has an action planning chapter, might work well for this stage. The Community Toolbox is an online resource that was developed to provide content, from assessment to evaluation, to help individuals learn community-building skills and engage in the process of taking action on community issues for social change (Center for Community Health and Development [CCHD], 2017; Schober & Fawcett, 2015). The integration of the action planning provided guidance about how to break large ideas into smaller parts (CCHD, 2017; Schober & Fawcett, 2015).

To use the Community Toolbox effectively, before the next session, the facilitators met and discussed how to integrate action planning into their facilitation with the youth participants. During Session 5, the facilitators introduced the action planning process to the youth. The facilitators asked the youth practical questions to make their solutions attainable, such as inquiring about who will carry out the action, when and where the action would take place, the length of time needed, necessary resources, and communication structures (CCHD, 2017). Through this process, the youth identified key stakeholders and resources and drafted the steps they would need to take in order to implement their action plans.

The facilitators continued to probe the youth about their action plans by asking them questions such as if the solution was focused and if the solution was attainable. Youth also had to identify the target for their solution, specifically which stakeholders in the community will help them carry out the plan. Youth also worked on identifying a rough time line. Youth also spent considerable time, over two sessions, discussing how they would implement the solution they set forth. They drafted potential activities for the community and village leaders that would help them progress toward achieving their goal. Also, as they began to see their action plans take shape, youth became visibly more comfortable with the process as they engaged with each other more and generated new ideas. Over the two sessions that youth worked on the action plans, the older youth tended to emerge into leadership and helped to drive the direction of the project, while their younger counterparts quietly contributed their ideas, playing a supportive role as they observed their peers. During this time, they still contributed to the crafting of the action plans.

### Session 7: Practice Presentation

Youth transitioned from developing the action plan to preparing for the community meeting. Before the final exhibit, youth self-selected presenters and practiced their presentations the day prior to the event. The older youth emerged as the speakers for each of their respective groups. As part of practicing their presentations, youth reviewed the action plan in front of their peers and facilitators, who then provided feedback and guidance for the final community meeting.

### Session 8: Community Meeting

**Figure 2.**
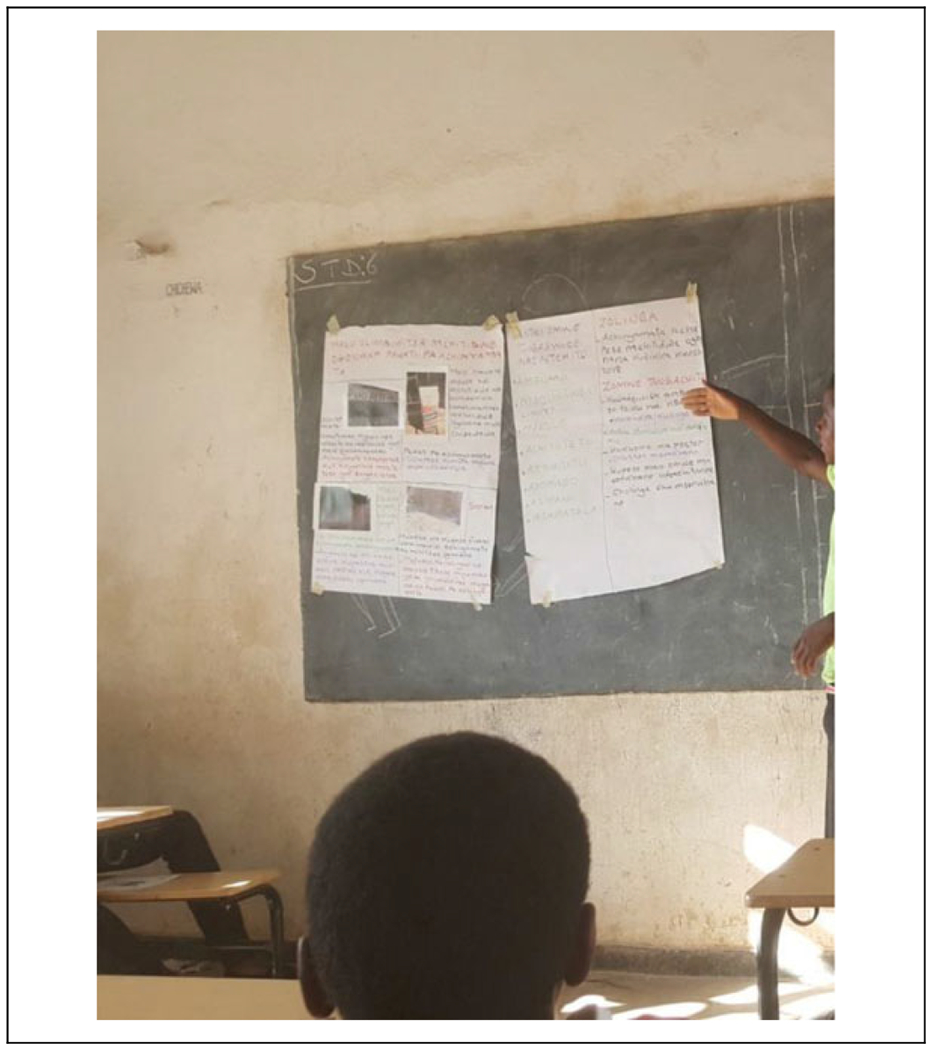
Exhibit of action plans—photographs and action plans presented together at the community exhibit.

For the final exhibit, youth presented their action plans and photographs to community leaders, elders, and parents (see Figure 2). Each person was able to take a role at the community event. Those who were less outspoken helped with setup and support throughout the presentations. The photographs illustrated the places and situations that they had identified relating to risky sexual behaviors. Community leaders also spoke at the celebratory event and recognized the work of the youth and the action plans. A small celebration of their achievement followed the presentation. As the next step in this research, at the end of the presentation, youth invited their parents and leaders for support to implement their action plans. Youth and facilitators ended the event with a plan to meet and evaluate the experience.

### The Action Plans Developed Through Photovoice

Youth identified assets that were protective as well as challenges in their environment that put them at risk; however, they decided to focus on the challenges for their action plans. After considerable discussion, the youth selected five themes to focus on for action plan development: initiation ceremonies; isolated places; community celebrations such as marriages, night dances, and sports events; local businesses such as bars and places selling alcohol as well as rest houses (motels) and video stores; and church-sponsored activities. The Youth Photovoice goal was to identify places and situations in the community associated with risky sexual behaviors of youth. Consequently, no action plans focused on individual factors such as deciding to abstain, using a condom, limiting partners, or a more careful selection of partners. Table 3 presents the problems youth identified and the solutions they came up with to address each of these problems. These plans were presented at the final exhibit. Below we provide more detail about each action plan.

*Initiation ceremonies* are a rite of passage for young men and women in Malawi as they transition from childhood to adulthood. In the male youth group, they noted that during the initiation ceremonies, youth are provided traditional medicine to arouse them sexually and told they should go out and have sexual intercourse. In the past, the initiation ceremonies were conducted around the age of 18, but as one young man noted, the ages for initiation ceremonies have dropped and now can take place with some participants as young as 5 years old. In the female youth group, it was suggested that counselors who over-see the ceremonies should avoid making remarks that may encourage youth to engage in sex. They also stated that young people should not engage in “dust cleaning,” which is a cultural practice where girls are expected to engage in sex to get cleansed as a final rite of passage (Gondwe, 2008).

*Celebratory and sporting events* were featured in the action plans as well. Celebratory events are special occasions. Youth particularly noted weddings but also discussed other general celebrations in the community. Sporting events, such as football (American soccer), occurred after school. In the boys’ group, they discussed the opportunities that exist while traveling to the game and after the game. One young man also noted that young men could become celebrities if they play well, which attracts young girls.

Local businesses, including *liquor stores, rest houses (motels), video shows, and bars*, were places the youth linked together as places that provided opportunities for risky sexual behaviors. In developing an action plan for addressing risky behavior, these were the local businesses that they chose to focus on. In the girls’ group, youth presented photographs of local bars and noted that young girls felt compelled to engage in sex with men for money to get uniforms to go to school and to have money for food during the school day. The girls’ group also noted that these places, particularly the bars, put young girls at risk because men can get drunk and forget to use a condom. It was also noted that youth were in bottle stores instead of school. Youth noted that some video shows would feature pornographic material. Rest houses were seen as places that youth can go to have sex.

*Isolated places* were identified as risky because isolation created opportunities for sexual activity. Encounters might be planned or accidental, or they may be an aggressive act. The youth linked power outages to increased risk because they would have to go to the maize mill when the electricity was turned back on; often, this was in the early and late evening.

*Religious organizations and activities* were viewed as both a protective and a risk factor for the youth. Because the area where Youth Photovoice occurred was predominantly Christian, none of the youth participants were Muslim and their commentary all used the term “church.” The youth felt that religious messages discouraging risky sexual behaviors were protective. However, they noted the opportunity to engage in risky sexual behavior after church events. It was suggested that leaders avoid organizing night prayers and could instead hold prayers during the day. Youth stated that the responsibility for such changes lies with adults, including the village chiefs, church elders, teachers, church priests, and parents.

## Discussion

This Youth Photovoice pilot with youth living in rural Malawi was successful in moving youth through the integrated photovoice action planning process aimed at making community-wide social change. Initially, youth were not familiar with the term “photovoice” or the action planning process. However, they rapidly gained an understanding of photovoice and action planning. The youth were able to take photographs and then discuss and analyze the photographs. Initially, getting from the analysis to action plans was challenging. Adding a structured action planning process was helpful for the facilitators as well as the youth. Youth were then able to use the structured action planning process to develop action plans for their setting. Through the integrated photovoice and action planning process, the youth were able to convert their experiences about places and situations that they associated with risk into succinct plans to present to community adults (parents and leaders). At the presentation, youth made plans to follow through with the implementation of these action plans in collaboration with parents, local leaders, and other community members.

Photovoice is well suited to facilitating sensitive topics with youth, such as discussions about sexual behavior because it does not require disclosure of one’s own experience. Instead, the process allows the youth to act as observers of their peers’ experiences. This is an important facet of the process: Since youth already face difficulties in communicating with their parents and other adults because of their age and stage of development, photovoice eliminates any awkward conversations between youth and parents and other adults that youth may try to avoid (Steinberg & Morris, 2001). This is amplified when discussing or attempting to discuss socially disapproved behaviors. Photovoice creates an opportunity for safe and open discussions that do not subject youth to judgment because the discussions do not require disclosing too much about oneself or owning the behavior that is being discussed.

The visual approach, taking photographs and discussing them, is highly engaging for youth and helps to build engagement. Initially, the youth were reticent about participating in the discussion because they were unfamiliar with one another. Group cohesion and engagement built over the sessions. Although focus group discussions could include up to 12 individuals, the use of a smaller group size of 6 encouraged full participation and reduced the likelihood that the younger ones would be excluded. The facilitator also modeled group inclusiveness by ensuring opportunities for each youth to participate in the analysis of the photographs and development of the action plan. Providing these opportunities allowed the younger members to open up and provide valuable insights to the discussions.

Engagement grew over time, and youth began to take on supportive and leadership roles in their groups. These roles became obvious as the youth began to develop their action plans. In addition, the research team observed that the older youth emerged as leaders during the action plan development, while their younger counterparts followed. This challenge is not unique to our project and has been noted elsewhere (Cubilla-Batista et al., 2017). One benefit of multiage groups is the opportunity to learn from their older peers; they observe leadership and benefit from observing leadership and potentially model such behavior among their age-mates or seek out leadership roles in the future. Facilitators also observed that older adolescents showed growing confidence with this leadership.

Photovoice also builds participants’ skills such as team building, social skill development, democratic decision making, and reflection (Wilson et al., 2007). Skill development was observed throughout the project by the facilitators and the adults who attended the community exhibit and presentation. Throughout the project, the youth became increasingly adept at communicating ideas. By the end of the photovoice process, the participants had a clear sense of connectedness to the project and their action plans. This growth in personal skills also is not unique to our project and adds support to what others have observed about the photovoice process (Cubilla-Batista et al., 2017; Wilson etal., 2007).

Facilitators played a key role in this project. Facilitators provided the action planning framework and helped youth fill in the content to complete the action plans. They needed to strike a delicate balance, helping the youth to move the project forward while ensuring that all youth voices were heard. Also, as the photovoice sessions progressed, facilitators became more aware of when to probe and when to hold back during the discussion. Also, they became more experienced in finding ways to intentionally involve youth throughout the process by setting clear objectives, as recommended for working with youth by Perez et al. (2016) and Wilson et al. (2007). After the facilitators’ experience in the pilot, they requested formal training that would enable them to sustain Youth Photovoice. The research team plans to develop such training based on the pilot experience.

Providing concrete steps in action plan development through the action plan module from the Community Toolbox helped youth visualize how to move from themes to action plans. Initially, the youth had difficulty moving from the analysis of the problem to the generation of specific action plans. Once the adapted action planning process was added, the youth were able to make this transition. This follows the recommendation of Perez et al. (2016) to have a structured process for the achievement of shared goals between the youth and the community.

The action plan module we integrated with photovoice complemented other commonly used photovoice analysis techniques such as SHOWeD and pile sorts. Having concrete action plans made it easier to visualize how the plans could be implemented for youth, their parents, and local leaders who attended the action plan presentation. Having photographs displayed next to the action steps was critical for visualization and provided context to the issues that the youth chose to target. The photographs were accessible to people, regardless of language barriers, education levels, or status. Presenting the photographs and the action plans to parents and local leaders opened a conversation between youth and adults about community changes that highlight the power of photovoice.

However, even though the action planning process added structure, the process could be seen as constraining. The focus on structure in the process may create a linear approach to engaging in community action, as opposed to recognizing the complexities of engaging in community work. In that photovoice is rooted in reflection, as evidenced by its underpinning frameworks of critical pedagogy and feminist theory, the action planning process may be too inflexible in its ability to provide opportunities for this reflection. It is also important to ensure that the action planning process does not compromise the empowerment of participants. Having a voice must stay at the center of the photovoice and action planning process. Therefore, when pairing the action planning process with photovoice, space must be created for an iterative process to occur where reflection and structure are equally encouraged (Liebenberg, 2018).

The Youth Photovoice pilot and the development of action plans proved to be highly appropriate for youth in rural Malawi. First, the youth commitment to this project was remarkable. Although they had additional responsibilities for school and their families, the youth would come, walking and biking from different neighborhoods, to the community center, on time for each day they were to be in attendance. Second, for the youth involved, this project provided new avenues of support from peers and community leaders. Often youth, particularly those who are not in school, have less access to supportive networks. Also, young women are more likely to be isolated and have limited social interactions outside of their family and close friends (Rock et al., 2016). In Youth Photovoice, young women and men engaged in this process separately and together. Moreover, the young women brought many ideas together to create the action plans that were subsequently presented to the community. Third, as in many African countries, age-based hierarchical relationships limit open communication between youth and adults. When youth attempt to discuss sexual issues with parents, this often results in punitive parental responses rather than open communication (Bastien et al., 2011; Steinberg & Morris, 2001; Wamoyi et al., 2010). Photovoice provided a vehicle for increasing youth communication with community adults, including their parents, without negative consequences for youth. The action plans that the youth developed and presented to local leaders and parents provided new insights for these adults and enhanced adults’ respect for the ideas of youth. Fourth, in this community, the local leaders, including village headmen and religious and educational leaders, have substantial authority to regulate the way things are done in their community. This means that local practices that youth identified as potentially increasing their risk can be changed at the community level.

### Limitations

Like most single-site qualitative studies, the Youth Photovoice pilot has limited generalizability. The community leadership structure in place in Malawi makes decision makers identifiable, and local leaders have the authority to make changes locally. Future research is needed to examine transferability to other countries and urban settings without this leadership. Also, this project was developed within the context of a community-led HIV prevention initiative for individual-level change, which had been collaborating with the research team for over 2 years. Although youth did not determine the focus of the Youth Photovoice pilot, community leaders had agreed that HIV prevention was a high priority for their community and that photovoice for youth should focus on community places and situations that affected youth HIV risk. Community members were already familiar with the facilitators. These established working relationship and trust facilitated the introduction of a novel project with youth that in some ways challenged the authority of parents and elders. Results may not be generalizable to other settings (or countries and urban areas) that do not have a similar community leadership structure and ongoing collaboration that has established trust.

Two other limitations should be considered. The focus on community-level factors limited the potential to develop action plans focused on individual-level factors. Future photovoice projects focused on HIV prevention with youth in Malawi could expand the focus. Additionally, only two coinvestigators had experience using photovoice. The lack of experience on the part of the facilitators may have hindered or stifled the voices of the youth, which might not have otherwise occurred with more experienced facilitators. To partially compensate for this lack of experience, the facilitators met before and after each session to discuss the process and ways to enhance their facilitation skills.

## Implications

Community-based participatory research has become recognized as a critical pathway toward HIV prevention (Campbell et al., 2013). However, it has seldom included youth in developing prevention initiatives meant explicitly for them. This successful Youth Photovoice pilot demonstrates that photovoice can engage youth and help them develop youth-led action plans to make changes in communities to reduce HIV risk specific to youth. Moreover, this visual approach may offer a strategy to overcome barriers in communicating about sexual behaviors. The proposed changes can have a direct impact on reducing youth risk for HIV infection by decreasing community situations that put them at risk for unsafe sex. The action plans not only have the potential to reduce situations related to unsafe sex, but they also have the potential to provide an opportunity to build upon assets within the community.

The Youth Photovoice pilot also has methodological implications for photovoice. The small addition to the photovoice process of engaging in a detailed action planning process can enhance the facilitation of solutions that have the potential to lead to community-wide changes. This technique has high potential to enhance action plan development within photovoice for many different community issues in many different settings.

## Figures and Tables

**Table 1.** Photovoice Sessions and Strategy Used to Advance Analysis.

Sessions	Activities	Discussion Elicitation Strategy
Session 1	Introduction and photovoice training	
Sessions 2 and 3	Facilitated discussion photos	SHOWeD discussion guide
Sessions 4 and 5	Identify issues and select photos	Pile sorting
	Develop and prioritize solutions	Strategy added by research team
Session 6	Develop action plans using Community Toolbox action plans	Community Toolbox—action plans
Session 7	Practice community presentation	
Session 8	Community presentation to parents and local stakeholders	Initial community mobilization—reaching local stakeholders

**Table 2.** Action Planning Process.

Action	Planning Process
Step 1	Categorize photographs into clusters through the pile sort process and develop a list of clusters.
Step 2	Provide details on each cluster, allowing each cluster to become a theme.
Step 3	Develop one solution for each theme identified.
Step 4	Prioritize and rank themes and paired solutions.
Step 5	Select the most pressing issue (theme and paired solution).
Step 6	Write out the solution to the issue and now brainstorm on how to expand upon it. Make sure to answer the following questions:
	What action will occur?
	Who will carry it out?
	When will it take place?
	How much time will it take?
	What resources are needed to carry out the action?
	What needs to be communicated?
	(Center for Community Health and Development, 2017)

**Table 3.** Action Plans the Youth Developed.

Issues Identified	Problems as Presented by Youth	Suggested Youth Action Plans
Initiation ceremonies	Youth discussed how part of this rite of passage emphasized sexual debut and encouraged “trying out” having sex.	• Increasing the age of initiation to 18 • Meet with community leaders to discuss this proposal
Isolated spaces	Isolated spaces put youth at risk of planned or unintended sexual activity, particularly with young people going out late at night.	• Meet with community to describe how going out late or at night creates the opportunity for sexual activity • Educate the public through posters • Develop skits to emphasize importance of walking in pairs and doing activities earlier in the day
Community celebrations—marriages, night dances, football games	Community celebrations, games, and night dances often include unsupervised time for meeting up with partners and engaging in sex.	• Adult presence at events • Encouraged parents to not let their children engage in night celebrations • Youth walk in pairs and that adults come to sporting events as they end • Suggested time changes so that sporting events held earlier in the evening • Meet with the police forum (community-led policing)
Local businesses attracting youth—rest houses, video shows, and bottle stores	These businesses provided access to risky activities including alcohol consumption and transactional sex.	• Advocated for police and village chiefs to be involved in regulating bottle stores (bars) and rest houses (short-term room rentals) • Role-plays as a medium to deliver message for youth to attend school
Church-sponsored activities	These events provided unintended opportunities for unsupervised meetings that put youth at risk.	• Holding prayers during the day and asked for leadership to step in with these events • Meeting with priests and/or church leaders to discuss timing of prayers and church events
